# Supplementary material for: Heartburn-Related Internet Searches and Trends of Interest across Six Western Countries: A Four-Year Retrospective Analysis Using Google Ads Keyword Planner
Source: Int J Environ Res Public Health. 2019 Nov 20;16(23):4591. doi: 10.3390/ijerph16234591 (PMC6926592; doi:10.3390/ijerph16234591)
Supplement: Supplementary file 1 [file ijerph-16-04591-s001.pdf]

Supplementary

Table S1. The top 20 keywords for each analyzed countries. Data presented as total number of queries of each keyword (percentage of total number of all keywords in the country).

Top keyword	Australia	Canada	Germany	Poland	The United Kingdom	The United States
**1.**	heartburn<br>2,194,500 (18.6%)	acid reflux<br>2,640,000 (13.3%)	sodbrennen<br>2,640,000 (8.9%)	refluks<br>2,640,000 (13.8%)	heartburn<br>2,640,000 (8.0%)	acid reflux<br>26,400,000 (13.5%)
**2.**	reflux<br>511,500 (4.3%)	gerd<br>2,640,000 (13.3%)	reflux<br>2,640,000 (8.9%)	zgaga<br>2,491,500 (13.0%)	acid reflux<br>2,640,000 (8.0%)	gerd<br>26,400,000 (13.5%)
**3.**	acid reflux<br>363,000 (3.1%)	heartburn<br>2,590,500 (13.0%)	was hilft gegen sodbrennen<br>2,244,000 (7.5%)	refluks żołądka<br>759,000 (4.0%)	gerd<br>2,640,000 (8.0%)	heartburn<br>24,420,000 (12.5%)
**4.**	heartburn symptoms<br>264,000 (2.2%)	heartburn symptoms<br>264,000 (1.3%)	hausmittel gegen sodbrennen<br>1,699,500 (5.7%)	refluks żołądkowo przełykowy<br>561,000 (2.9%)	reflux<br>2,541,000 (7.7%)	heartburn symptoms<br>2,640,000 (1.3%)
**5.**	gerd<br>264,000 (2.2%)	reflux<br>264,000 (1.3%)	sodbrennen schwangerschaft<br>1,254,000 (4.2%)	refluks objawy<br>363,000 (1.9%)	what causes heartburn<br>2,244,000 (6.8%)	reflux<br>2,640,000 (1.3%)
**6.**	what causes heartburn<br>264,000 (2.2%)	what causes heartburn<br>264,000 (1.3%)	refluxösophagitis<br>1,155,000 (3.9%)	zgaga w ciąży<br>264,000 (1.4%)	silent reflux<br>858,000 (2.6%)	what causes heartburn<br>2,640,000 (1.3%)
**7.**	heartburn remedies<br>264,000 (2.2%)	heartburn remedies<br>264,000 (1.3%)	sodbrennen was hilft<br>966,900 (3.2%)	refluks dieta<br>264,000 (1.4%)	acid reflux symptoms<br>759,000 (2.3%)	heartburn medicine<br>2,640,000 (1.3%)
**8.**	gerd symptoms<br>264,000 (2.2%)	acid reflux symptoms<br>264,000 (1.3%)	sodbrennen hausmittel<br>957,000 (3.2%)	co na za zgage<br>264,000 (1.4%)	gerd symptoms<br>561,000 (1.7%)	heartburn remedies<br>2,640,000 (1.3%)
**9.**	silent reflux<br>264,000 (2.2%)	gerd diet<br>264,000 (1.3%)	speiseröhrenentzündung<br>610,500 (2.0%)	Lek na zgage<br>264,000 (1.4%)	heartburn symptoms<br>412,500 (1.2%)	acid reflux symptoms<br>2,640,000 (1.3%)
**10.**	reflux symptoms<br>264,000 (2.2%)	gerd symptoms<br>264,000 (1.3%)	refluxkrankenheit<br>462,000 (1.5%)	sposób na zgagę<br>264,000 (1.4%)	heartburn remedies<br>363,000 (1.1%)	acid reflux medicine<br>2,640,000 (1.3%)

**11.**	heartburn pregnancy 264,000 (2.2%)	heartburn home remedy 264,000 (1.3%)	sodbrennen in der schwangerschaft 462,000 (1.5%)	domowe sposoby na zgage 264,000 (1.4%)	how to get rid of heartburn 313,500 (0.9%)	acid reflux diet 2,640,000 (1.3%)
**12.**	how to get rid of heartburn 264,000 (2.2%)	what causes acid reflux 264,000 (1.3%)	sodbrennen symptome 313,500 (1.0%)	sposoby na zgage 259,050 (1.4%)	what is heartburn 264,000 (0.8%)	gerd diet 2,640,000 (1.3%)
**13.**	acid reflux symptoms 259,050 (2.2%)	gastroesophageal reflux disease 264,000 (1.3%)	speiseröhre 313,500 (1.0%)	refluks u niemowlaka 259,050 (1.4%)	acid reflux diet 264,000 (0.8%)	gerd symptoms 2,640,000 (1.3%)
**14.**	gastroesophageal reflux disease 259,050 (2.2%)	how to get rid of heartburn 264,000 (1.3%)	reflux symptome 288,750 (1.0%)	co na zgagę w ciąży 254,100 (1.3%)	gerd diet 264,000 (0.8%)	heartburn home remedy 2,640,000 (1.3%)
**15.**	what is heartburn 229,350 (1.9%)	what does heartburn feel like 264,000 (1.3%)	mittel gegen sodbrennen 264,000 (0.9%)	dieta przy refluksie 249,150 (1.3%)	acid reflux treatment 264,000 (0.8%)	what causes acid reflux 2,640,000 (1.3%)
**16.**	what is heartburn 229,350 (1.9%)	acid reflux diet 259,050 (1.3%)	gegen sodbrennen 264,000 (0.9%)	refluks u dzieci 234,300 (1.2%)	heartburn home remedy 264,000 (0.8%)	gastroesophageal reflux disease 2,640,000 (1.3%)
**17.**	what is reflux 204,600 (1.7%)	home remedies for acid reflux 259,050 (1.3%)	hausmittel sodbrennen 264,000 (0.9%)	zapalenie przełyku 234,300 (1.2%)	constant heartburn 264,000 (0.8%)	what is gerd 2,640,000 (1.3%)
**18.**	heartburn home remedy 199,650 (1.7%)	heartburn pregnancy 259,050 (1.3%)	was hilft bei sodbrennen 264,000 (0.9%)	objawy refluksu 229,350 (1.2%)	what is acid reflux 264,000 (0.8%)	home remedies for acid reflux 2,640,000 (1.3%)
**19.**	what causes acid reflux 165,000 (1.4%)	natural remedies for heartburn 249,150 (1.3%)	sodbrennen ursachen 264,000 (0.9%)	na zgage 224,400 (1.2%)	heartburn cure 264,000 (0.8%)	how to get rid of heartburn 2,640,000 (1.3%)
**20.**	reflux treatment 120,450 (1.0%)	what is heartburn 244,200 (1.2%)	was tun bei sodbrennen 264,000 (0.9%)	zgaga obawy 214,500 (1.1%)	what causes acid reflux 264,000 (0.8%)	what does heartburn feel like 2,640,000 (1.3%)

Table S2. Comparison between the number of searches per month in the Google search engine of categories of keywords associated with heartburn in the analyzed countries in each season (1a) and each year (1b). Data are presented as median (interquartile range).

A) Category of keyword: Treatment.

Country	Spring (Sp)	Summer (Su)	Fall (F)	Winter (W)	Differences between Seasons	Post Hoc Test
**Australia**	56,838 (52,655–66,953)	57,258 (47,959–60,134)	51,023 (41,745–53,599)	56,860 (46,783–61,489)	H(3) = 6.73; $p = 0.08$	-
**Canada**	97,933 (88,119–100,580)	79,540 (72,583-87,015)	87,535 (76,213–98,775)	92,435 (88,300–99,704)	H(3) = 12.09; $p < 0.01$	Su vs. W: $p = 0.007$
**Germany**	255,805 (226,010–298,675)	190,113 (180,976–213,110)	258,840 (234,233–314,674)	283,130 (260,119–333,581)	H(3) = 19.11; $p < 0.001$	Sp vs. Su: $p < 0.01$; Su vs. F: $p = 0.004$; Su vs. W: $p < 0.001$
**Poland**	99,715 (90,533–117,684)	81,820 (69,785–88,553)	101,053 (90,823–113,710)	118,873 (112,256–128,053)	H(3) = 21.76 ; $p < 0.001$	Sp vs. Su: $p = 0.009$; Sp vs. W: $p = 0.03$; Su vs. F: p =0.009; Su vs. W: $p < 0.001$; A vs. W: $p = 0.03$
**United Kingdom**	140,608 (138,900–143,379)	121,030 (93,853–132,338)	136,723 (105,770–141,623)	142,835 (128,020–149,459)	H(3) = 12.80; $p < 0.01$	Sp vs. Su: $p = 0.009$; Su vs. W: $p = 0.02$
**United States**	952,813 (898,190–980,445)	876,163 (799,720–943,704)	849,210 (785,243–984,876)	952,565 (884,305–1,051,256)	H(3) = 6.41; $p = 0.09$	-

Categories of Keywords	1st year	2nd year	3rd year	4th year	Differences between Seasons	Post Hoc Test
Australia	42,183 (40,031–51,455)	51,513 (44,508–55,759)	55,575 (52,419–60,005)	63,793 (59,525–67,174)	H(3) = 24.35; $p < 0.001$	1st vs. 3rd: $p < 0.006$; 1st vs. 4th: $p < 0.001$; 2nd vs. 4th: $p < 0.001$; 3rd: 4th: $p = 0.02$
Canada	76,298 (72,446-85,629)	88,078 (80,011–93,018)	92,783 (88,428–98,095)	103,158 (97,240–108,195)	H(3) = 26.30; $p < 0.001$	1st vs. 2nd: $p = 0.03$; 1st vs. 3rd: $p < 0.001$; 1st vs. 4th: $p < 0.001$; 2nd vs. 4th: $p = 0.001$; 3rd vs. 4th: $p = 0.02$
Germany	237,413 (215,499–250,976)	235,035 (208,398–265,348)	274,220 (245,446–296,864)	324,783 (217,220–335,020)	H(3) = 7.63; $p = 0.05$	-
Poland	83,645 (70,858–98,720)	97 538 (84 999-104 951)	108 328 (90 533-121 520)	122 433 (109 848.8-133 424)	H(3)=16.61; $p < 0.001$	1st vs. 3rd: P=.02; 1st vs. 4th: P=.002; 2nd vs. 4th: P=.009
United Kingdom	101 203 (96 824-114 879)	134,298 (116,563–140,484)	140,680 (138,281–142,525)	143,083 (140,174–144,481)	H(3) = 2.04; $p < 0.001$	1st vs. 2nd: $p = 0.04$; 1st vs. 3rd: $p < 0.001$; 1st vs. 4th: $p < 0.001$; 2nd vs. 4th: $p = 0.02$
United States	814,138 (788,026–871,758)	852,650 (831,514–944,373)	946,328 (908,931–966,240)	1,022,335 (978,106–1,069,459)	H(3) = 28.00; $p < 0.001$	1st vs. 3rd: $p = 0.001$; 1st vs. 4th: $p < 0.001$; 2nd vs. 4th: $p < 0.001$; 3rd vs. 4th: $p < 0.001$

B) Category of keyword: Home-based treatment.

**Country**	**Spring (Sp)**	**Summer (Su)**	**Fall (F)**	**Winter (W)**	**Differences between seasons**	**Post hoc test**
Australia	7425 (7301–7920)	6930 (5198–7425)	2228 (1856–7425)	7425 (5816–7536)	H(3) = 6.39; p = 0.09	-
Canada	12,623 (12,375–12,870)	12,375 (12,375–12,499)	12,623 (12,375–12,870)	12,623 (12 375–12,870)	H(3) = 3.56; p = 0.31	-
Germany	86,418 (70,390–130,370)	32,758 (31,173–79,459)	88,818 (69,238-104,794)	91,763 (82,828–108,854)	H(3) = 13.17; p < 0.01	Sp vs. Su: p = 0.01; Su vs. F: p = 0.03; Su vs. W: p = 0.004
Poland	22,418 (16,923–25,301)	15,933 (12,344–18,210)	23,160 (17,120–25,759)	26,403 (25,474–27,133)	H(3) = 23.53; p < 0.001	Sp vs. Su: p = 0.01; Sp vs. W: p = 0.01; Su vs. F: p = 0.01; Su vs. W: p < 0.001; F vs. W: p = 0.01
United Kingdom	12,870 (12,375–12,870)	7178 (6930–12,870)	12,870 (7301–12,870)	12,623 (11,261–13,365)	H(3) = 7.91; p = 0.048	Sp vs. Su: p = 0.048; Su vs. W: p = 0.048
United States	128,700 (123,750–128,700)	126,225 (123,750–128,700)	128,700 (128,700–128,700)	128,700 (123,750–141,075)	H(3) = 3.10; p = 0.38	-

Categories of Keywords	1st year	2nd year	3rd year	4th year	Differences between Seasons	Post Hoc Test
Australia	1485 (1485–6435)	6683 (2351–7425)	7648 (7425–7920)	7425 (7425–7920)	H(3) = 29.41; $p < 0.001$	1st vs. 2nd: $p = 0.006$; 1st vs. 3rd: $p < 0.001$; 1st vs. 4th: $p < 0.001$; 2nd vs 3rd: $p = 0.005$; 2nd vs. 4th: $p = 0.006$
Canada	12,375 (12,251–12,375)	12,375 (12,375–12,375)	12,870 (12,746–12 870)	12,870 (12,870–12,870)	H(3) = 36.82; $p = 0.001$	1st vs. 3rd: $p < 0.001$; 1st vs. 4th: $p < 0.001$; 2nd vs. 4th: $p < 0.001$; 3rd vs. 4th: $p < 0.001$
Germany	81,118 (35,800–96,511)	60,800 (37 126–95 116)	90,873 (81 516–131 065)	90,080 (37,275–97,259)	H(3) = 3.08; $p = 0.38$	-
Poland	17,145 (15,301–24,249)	20,958 (16,403–24,769)	25,115 (17,690–27,170)	25,635 (20,933–26,625)	H(3) = 8.78; $p = 0.03$	-
United Kingdom	7178 (6930–7920)	12,375 (7301–12,499)	12,870 (12,870–13,365)	12,870 (12,870–13,489)	H(3) = 29.10; $p < 0.001$	1st vs. 2nd: $p = 0.03$; 1st vs. 3rd: $p < 0.001$; 1st vs. 4th: $p < 0.001$; 2nd: 3rd: $p < 0.01$; 2nd vs. 4th: $p < 0.01$
United States	126,225 (123,750–128,700)	123,750 (123,750–123,750)	128,700 (128,700–128,700)	128,700 (128,700–178,200)	H(3) = 25.06; $p < 0.001$	1st vs. 3rd: $p = 0.040$; 1st vs. 4th: $p = 0.003$; 2nd vs. 3rd: $p = 0.003$; 2nd vs. 4th: $p < 0.001$; 3rd vs. 4th: $p = 0.040$

C) Category of keyword: Herbal remedies.

Country	Spring (Sp)	Summer (Su)	Fall (F)	Winter (W)	Differences between seasons	Post hoc test
Australia	6385 (3465–8860	3465 (3291–7883)	3390 (2920–3465)	3365 (2759–4850)	H(3) = 12.00; $p < 0.01$	Sp vs. Su: $P = 0.01$; Sp vs. W: $P = 0.03$;
Canada	9900 (9776–9900)	9158 (8736–9405)	9405 (9405–9900)	9900 (9776–9900)	H(3) = 17.63; $p < 0.001$	Sp vs. Su: $p = 0.004$; Su vs. W: $p < 0.004$
Germany	6710 (5991–7155)	5620 (5125–6040)	6090 (5620–7650)	7155 (6635–7700)	H(3) = 13.44; $p < 0.01$	Sp vs. Su: $p = 0.04$; Su vs. W: $p = 0.004$
Poland	4543 (4283–5185)	3800 (3700–4295)	4295 (4170–4456)	4840 (4740–5866)	H(3) = 21.26; $p < 0.001$	Sp vs. Su: $p = 0.005$; Su vs. F: $p = 0.02$; Su vs. W: $p < 0.001$; F vs. W: $p = 0.02$
United Kingdom	15,345 (14,850–15,469)	14,330 (9900–14,850)	15,320 (9405–15,456)	15,345 (14,800–15,456)	H(3) = 6.29; $p = 0.10$	-
United States	147,510 (98,010–147,510)	93 060 (88 110-98 134)	95,535 (76,973–98,134)	122,760 (96,773–152,460)	H(3) = 10.97; $p = 0.01$	-

Categories of Keywords	1st year	2nd year	3rd year	4th year	Differences between seasons	Post hoc test
Australia	3143 (2475–3564)	3415 (2920–4938)	3885 (3415–8315)	3465 (3403–5309)	H(3) = 3.77; $p = 0.29$	-
Canada	9405 (9269–9529)	9900 (9393–9900)	9405 (9405–9900)	9900 (9405–9900)	H(3) = 4.96; $p = 0.17$	-
Germany	5670 (5100–6078)	6413 (6090–7291)	6140 (5 608–7 241)	7180 (6574–7316)	H(3) = 11.64; $p < 0.01$	1st vs. 2nd: $p = 0.03$; 1st vs. 4th: $p = 0.0095$
Poland	4095 (3738–4295)	4568 (4146–4840)	4295 (4283–4753)	5433 (4295–5941)	H(3) = 11.96; $p < 0.01$	1st vs. 4th: $p = 0.01$
United Kingdom	9628 (9393–14,850)	15,098 (13,241–15,345)	15,320 (14,850–15,803)	15,345 (15,184–15,456)	H(3) = 13.93; $p < 0.01$	1st vs. 2nd: $p = 0.04$; 1st vs. 3rd: $p = 0.008$; 1st vs. 4th: $p = 0.008$
United States	95,535 (88,110–147,510)	93,060 (78,210–98,134)	145,035 (98,010–148,748)	98,010 (96,773–110,756)	H(3) = 5.35; $p = 0.15$	-

D) Category of keyword: Diet.

Country	Spring (Sp)	Summer (Su)	Fall (F)	Winter (W)	Differences between seasons	Post hoc test
Australia	13,310 (10,290–16,329)	10,588 (9,919–15,846)	10,538 (9,411–11,293)	10,563 (10,328–11,293)	H(3) = 2.68; $p = 0.44$	-
Canada	30,635 (25,561–32,355)	23,705 (21,478–25,314)	29,125 (24,324–30,128)	31,130 (26,353–3,231)	H(3) = 13.56; $p < 0.01$	Sp vs. Su: $p = 0.008$; Su vs. W: $p = 0.008$
Germany	67,075 (50,978–73,588)	48 065 (36,379–52,005)	61,533 (47,244–71,991)	69,728 (64,404–90,951)	H(3) = 15.68; $p < 0.01$	Sp vs. Su: p=.02; Su vs. F: $p = 0.04$; Su vs. W: p=.001
Poland	44,110 (41,590–46,015)	33,663 (29,421–40,705)	43,293 (37,418–46,389)	46,853 (44,520–56,374)	H(3) = 19.23; $p < 0.001$	Sp vs. Su: $p = 0.001$; Su vs. W: $p < 0.001$
United Kingdom	38,308 (35,585–43,629)	30,883 (29,645–35,956)	35,065 (30,635–37,813)	38,555 (35,833–39,669)	H(3) = 12.55; $p < 0.01$	Sp vs. Su: $p = 0.02$; Su vs. W: $p = 0.02$
United States	281,600 (247,321–313,775)	246,208 (237,298–294,594)	243,733 (237,050–305,236)	307,588 (285,684–319,715)	H(3) = 7.97; $p = 0.046$	-

Categories of Keywords	1st year	2nd year	3rd year	4th year	Differences between seasons	Post hoc test
Australia	8805 (7666–9350)	10,340 (10,290–10,959)	11,230 (10,340–15,748)	16,205 (14,571–16,366)	H(3) = 31.62; $p < 0.001$	1st vs. 2nd: $p < 0.001$; 1st vs. 3rd: $p < 0.001$; 1st vs. 4th: $p < 0.001$; 2nd vs. 4th: $p = 0.001$; 3rd vs. 4th: $p = 0.02$
Canada	23,210 (20,735–25,685)	28,655 (24,571–30,103)	29,645 (26,105–30,883)	32,813 (30,103–33,221)	H(3) = 19.16; $p < 0.001$	1st vs. 2nd: $p = 0.03$; 1st vs. 3rd: $p = 0.008$; 1st vs. 4th: $p = 0.003$; 2nd vs. 4th: $p < 0.02$; 3rd vs. 4th: $p = 0.03$
Germany	45,840 (41,168–53,249)	61,433 (48,436–66,943)	62,400 (50,768–70,295)	93,958 (70,494–95,505)	H(3) = 19.61; $p < 0.001$	1st vs. 2nd: $p = 0.03$; 1st vs. 3rd: $p = 0.03$; 1st vs. 4th: $p < 0.001$; 2nd vs. 4th: $p = 0.009$; 3rd vs. 4th: $p = 0.01$
Poland	39,473 (29,421–41,170)	42,055 (33,648–44,000)	45,620 (41,809–47,106)	47,773 (44,814–56,225)	H(3) = 19.09; $p < 0.001$	1st vs. 3rd: $p = 0.01$; 1st vs. 4th: $p < 0.001$; 2nd vs. 4th: $p = 0.004$
United Kingdom	30,635 (29,893–30,635)	34,843 (30,635–37,936)	37,813 (37,441–38,803)	38,555 (37,404–44,111)	H(3) = 24.79; $p < 0.001$	1st vs. 2nd: $p = 0.02$; 1st vs. 3rd: $p < 0.001$; 1st vs. 4th: $p < 0.001$
United States	238,783 (229,130–245,218)	243,980 (243,361–295,831)	297,935 (252,148–312,290)	320,458 (317,240–327,140)	H(3) = 27.72; $p < 0.001$	1st vs. 3rd: $p =0.004$; 1st vs. 4th: $p < 0.001$; 2nd: vs. 4th: $p < 0.001$; 3rd vs. 4th: $p = 0.004$

E) Category of keyword: What is heartburn?

Country	Spring (Sp)	Summer (Su)	Fall (F)	Winter (W)	Differences between seasons	Post hoc test
Australia	15,865 (15,703–17,461)	15,765 (15,308–16,285)	15,515 (14,081–15,915)	10,593 (5 579–15 331)	H(3) = 12.05; $p < 0.01$	Sp vs. W: $p = 0.01$
Canada	21,930 (16,955–22 129)	14,158 (9764–18,193)	21,558 (17,043–21,968)	21,930 (17,501–22,216)	H(3) = 12.81; $p < 0.01$	Sp vs. Su: $p = 0.01$; Su vs. F: $p = 0.040$; Su vs. W: $p \leq 0.009$
Germany	8870 (8475–8970)	8500 (7880–8983)	8920 (8475–8970)	9020 (8821–9478)	H(3) = 7.67; $p = 0.05$	-
Poland	5418 (5133–10,244)	4378 (3956–5281)	4898 (4080–7150)	5170 (4650–10120)	H(3) = 5.58; $p = 0.13$	-
United Kingdom	28,715 (28,565–29,135)	27,895 (27,563–28,169)	28,640 (28,245–29,135)	28,615 (28,578–29,309)	H(3) = 13.60; $p < 0.01$	Sp vs. Su: $p = 0.008$; Su vs. F: $p = 0.02$; Su vs. W: $p = 0.008$
United States	201,003 (198,676–203,886)	196,745 (183,751–198,813)	199,023 (197,624–202,203)	200,483 (197,760–203,478)	H(3) = 9.77; $p = 0.02$	Sp vs. Su: $p = 0.03$

Categories of Keywords	1st year	2nd year	3rd year	4th year	Differences between seasons	Post Hoc Test
Australia	10,765 (5753–15,840)	15,640 (10,779–15,878)	15,640 (15,283–15,953)	16,013 (15,354–20,690)	H(3) = 7.33; $p = 0.06$	-
Canada	16,705 (10,148–17,018)	19,380 (14,651–22,325)	21,905 (20,221–22,005)	21,930 (21,793–22,204)	H(3) = 17.37; $p < 0.001$	1st vs. 3rd: $p = 0.003$; 1st vs. 4th: $p < 0.001$
Germany	8475 (7980–8475)	8525 (8326–9020)	8970 (8920–9131)	8945 (8870–9131)	H(3) = 20.94; $p < 0.001$	1st vs. 3rd: $p < 0.001$; 1st vs. 4th: $p <.001$
Poland	4080 (3956–4279)	4650 (4080–4799)	5665 (5170–10,120)	10,368 (6024–10,615)	H(3) = 32.68; $p < 0.001$	1st vs. 2nd: $p = 0.047$; 1st vs. 3rd: $p < 0.001$; 1st vs. 4th: $p < 0.001$; 2nd vs. 3rd: $p = 0.001$; 2nd vs. 4th: $p < 0.001$
United Kingdom	28,268 (27,859–28,590)	28,765 (28,416–29,346)	28,590 (28,429–29,160)	28,615 (28,095–29,296)	H(3) = 6.86; $p = 0.08$	-
United States	201,473 (146,131–202,488)	197,760 (197,290–201,844)	197,760 (197,154–198,825)	203,750 (199,641–207,981)	H(3) = 12.58; $p < 0.01$	2nd vs. 4th: $p = 0.005$; 3rd vs. 4th: $p = 0.005$

F) Category of keyword: Causes.

Country	Spring (Sp)	Summer (Su)	Fall (F)	Winter (W)	Differences between seasons	Post hoc test
Australia	20,715 (20,641–21,820)	20,790 (16,398–22 329)	19,100 (15,903–20,726)	20,315 (15,554–20,924)	H(3) = 6.55; *p* = 0.09	-
Canada	21,265 (20,979–22,221)	20,395 (19,643–20,816)	20,818 (20,175–21,440)	21,365 (21,145–21,651)	H(3) = 14.78; *p* < 0.01	Sp vs. Su: *p* = 0.006; Su vs. W: *p* = 0.006
Germany	26,048 (25,069–27,521)	18,450 (17,918–19,315)	25,875 (23,906–27,089)	26,815 (26,259–28,041)	H(3) = 26.31; *p* < 0.001	Sp vs. Su: *p* < 0.001; Su vs. F: *p* < 0.001; Su vs. W: *p* < 0.001
Poland	15,385 (14,071–20,223)	10,633 (9653–14,964)	15,285 (13,353–15,634)	20,335 (15,719–20,916)	H(3) = 17.13; *p* < 0.001	Sp vs. Su: *p* = 0.02; Su vs. F: *p* = 0.03; Su vs. W: *p* < 0.001; F vs. W: *p* = 0.045
United Kingdom	77,085 (76,005–82,196)	74,708 (26,343–75,450)	76,193 (76,083–76,515)	77,108 (76,960–77,580)	H(3) = 21.84; *p* < 0.001	Sp vs. Su: *p* = 0.006; Su vs. F: *p* = 0.006; Su vs. W: *p* < 0.001; F vs. W: *p* = 0.006
United States	171,610 (170,051–181,263)	170,150 (167,600–172,105)	170,695 (170,496–176,609)	171,585 (171,041–177,290)	H(3) = 5.34; *p* = 0.15	-

Categories of Keywords	1st year	2nd year	3rd year	4th year	Differences between seasons	Post hoc test
Australia	19,620 (15,575-20 454)	16,163 (15 391-20 590)	20,715 (20,565-20,839)	22,205 (21,743-22,513)	H(3)=20.48; *p* < 0.001	1st vs. 4th: *p* =0.002; 2nd vs. 3rd: *p* =.02; 2nd vs. 4th: *p*=0.002; 3rd vs. 4th: *P*=.002
Canada	20,078 (19,643-20,438)	21,115 (20,276-21,278)	21,340 (21,078-21,760)	21,490 (21,115-21,985)	H(3)=21.50; *p* < 0.001	1st vs. 2nd: *P*=0.03; 1st vs. 3rd: *p*<0.001; 1st vs. 4th: *P*<0.001; 2nd vs. 4th: *p*=0.049
Germany	24,588 (21,554-25,591)	25,578 (20,468-26,531)	25,750 (23,648-27,076)	27,658 (25,343-28,499)	H(3)=8.80; *p* =0.03	1st vs. 4th: *p*=0.049
Poland	11,228 (9578-16,460)	15,385 (14,828-15,893)	15,310 (10,669-20,793)	15,658 (15,185-20,384)	H(3)=5.21; *p* = 0.16	-
United Kingdom	76,093 (63,211-76,985)	75,848 (74,830-76,739)	76,290 (75,859-76,998)	77,258 (76,218-82,629)	H(3)=5.32; *p* = 0.15	-
United States	167,625	170,670	171,140	180,768	H(3)=22.65; *p* < 0.001	1st vs. 3rd: *p* = 0.02; 1st vs. 4th: *p*<0.001;

	(161,424-171,523)	(170,163-170,831)	(170,163-176,609)	(176,808-182,599)	2nd vs. 4th: $p < 0.001$; 3rd vs. 4th: p=0.02

G) Category of keyword: Symptoms

Country	Spring (Sp)	Summer (Su)	Fall (F)	Winter (W)	Differences between seasons	Post hoc test
Australia	34,000 (30,519-36,274)	30,410 (30,011-31,881)	30,358 (30,186-32,080)	31,128 (30,224-34,801)	H(3)=6.38; *p*=0.09	-
Canada	32,810 (32,270-33,555)	31,673 (31,164-32,008)	32,810 (31,798-33,146)	32,763 (32,158-33,430)	H(3)=9.53; *p*=0.02	Sp vs. Su: *p*=0.040; Su vs. W: *p*=0.03
Germany	14,330 (13,810-14,850)	13,860 (13,760-14,268)	14,305 (13,798-14,850)	14,305 (14,305-14,603)	H(3)=7.29; *p*=0.06	-
Poland	33,315 (24,183-38,548)	26,260 (20,864-31,606)	30,345 (28,686-32,408)	34,058 (32,545-36,236)	H(3)=10.99; *p*=0.01	Su vs. W: *p*=0.01
United Kingdom	70,140 (44,796-95,633)	44,993 (44,584-45,535)	68,135 (45,714-94,618)	45,688 (45,415-58,285)	H(3)=5.79; *p*=0.12	-
United States	295,393 (290,283-300,554)	290,740 (284,589-293,895)	292,125 (280,519-299,823)	300,145 (294,131-306,196)	H(3)=10.91; *p*=0.01	Su vs. W: *p*=0.03

**Categories of Keywords**	**1st year**	**2nd year**	**3rd year**	**4th year**	**Differences between seasons**	**Post hoc test**
Australia	29,840 (29,220-30,298)	30,383 (30,173-30,696)	30,805 (30,519-31,783)	35,728 (35,643-36,124)	H(3) = 27.34; $p < 0.001$	1st vs. 3rd: $p = 0.003$; 1st vs. 4th: $p < 0.001$; 2nd vs. 4th: $p < 0.001$; 3rd vs. 4th: $p < 0.001$
Canada	31,130 (30,856-32,045)	32,020 (31,689-32 875)	33,355 (32,998-33,505)	32,860 (32,365-32,960)	H(3) = 19.27; $p < 0.001$	1st vs. 3rd: $p = 0.001$; 1st vs. 4th: $p = 0.001$
Germany	14,255 (13,798-14,268)	14,058 (9900-14,318)	14,305 (14,156-14,454)	15 098 (14,701-15,444)	H(3) = 14.09; $p < 0.01$	1st vs. 4th: $p = 0.01$; 2nd vs. 4th: $p = 0.01$; 3rd vs. 4th: $p = 0.04$
Poland	30,935 (22,793-33,053)	28,340 (23,699-31,866)	32,078 (28,873-34,219)	34,330 (31,694-43,068)	H(3) = 9.50; $p = 0.02$	2nd vs. 4th: $p = 0.04$
United Kingdom	93,678 (45,391-94,149)	45,243 (43,658-46,134)	45,365 (44,721-56,836)	93,133 (45,474-95,595)	H(3) = 8.85; $p = 0.03$	-
United States	283,860 (278,378-288,971)	292,275 (289,936-301,433)	299,873 (294,501-302,484)	295,145 (292,794-295,986)	H(3) = 10.77; $p = 0.01$	1st vs. 3rd: $p =0.03$

H) Category of keyword: Pregnant

**Country**	**Spring (Sp)**	**Summer (Su)**	**Fall (F)**	**Winter (W)**	**Differences between seasons**	**Post hoc test**
Australia	10,175 (10,125-10,744)	10,125 (9630-10,620)	10,125 (9630-10,249)	10,125 (9668-10,286)	H(3) = 2.78; p = 0.43	-
Canada	11,115 (11,115-11,128)	11,115 (11,115-11,165)	11,115 (11,115-11,165)	11,140 (11,115-12,489)	H(3) = 2.88; p = 0.41	-
Germany	74,945 (27,190-81,850)	27,673 (25,953-75,601)	82 123 (37,251-84,610)	35,048 (31,150-76,690)	H(3) = 10.87; p = 0.01	Su vs. F: p = 0.007
Poland	20,370 (19,850-21,523)	20,445 (19,010-20,990)	21,260 (21,003-21,919)	21,560 (21,211-21,968)	H(3) = 15.13; p < 0.01	Sp vs. W: p = 0.03; Su vs. F: p = 0.03; Su vs. W: p = 0.004
United Kingdom	26,015 (25,965-26,460)	25,965 (25,965-26,015)	25,965 (24,740-26,126)	25,965 (24,728-26,126)	H(3) = 1.16; p = 0.76	-
United States	116,105 (111,155-130,955)	116,105 (111,155-160,655)	113,630 (111,155-130,918)	160,655 (116,105-165,605)	H(3) = 4.22; p = 0.24	-

Categories of Keywords	1st year	2nd year	3rd year	4th year	Differences between seasons	Post hoc test
**Australia**	9630 (9630-9680)	10,125 (10,125-10,175)	10,150 (10,125-10,620)	10,150 (10,125-10,620)	H(3) = 17.25; $p < 0.001$	1st vs. 2nd: $p = 0.005$; 1st vs. 3rd: $p = 0.005$; 1st vs. 4th: $p = 0.002$
**Canada**	11,115 (11,115-11,165)	11,140 (11,004-12,489)	11,115 (11,115-11,165	11,115 (11,115-11,115)	H(3) = 1.45; $p = 0.69$	-
**Germany**	55,120 (28,155-76,244)	78,385 (31,175-82,036)	37,078 (32,226-81,441)	33,613 (27,770-83,583)	H(3) = 0.96; $p = 0.81$	-
**Poland**	20,320 (19,528-21,260)	21,065 (20,458-21,523)	21,560 (20,558-22,129)	21,065 (20,854-21,485)	H(3) = 7.28; $p = 0.06$	-
**United Kingdom**	26,238 (25,965-26,473)	23,763 (21,015-25,965)	25,965 (25,965-25,978)	26,015 (25,965-26,460)	H(3) = 10.97; $p = 0.01$	1st vs. 2nd: $p = 0.04$; 1st vs. 3rd: $p = 0.044$
**United States**	118,580 (116,105-165,605)	118,555 (111,155-160,655)	111,155 (111,155-116,105)	160,655 (111,155-160,655)	H(3) = 7.61; $p = 0.05$	-

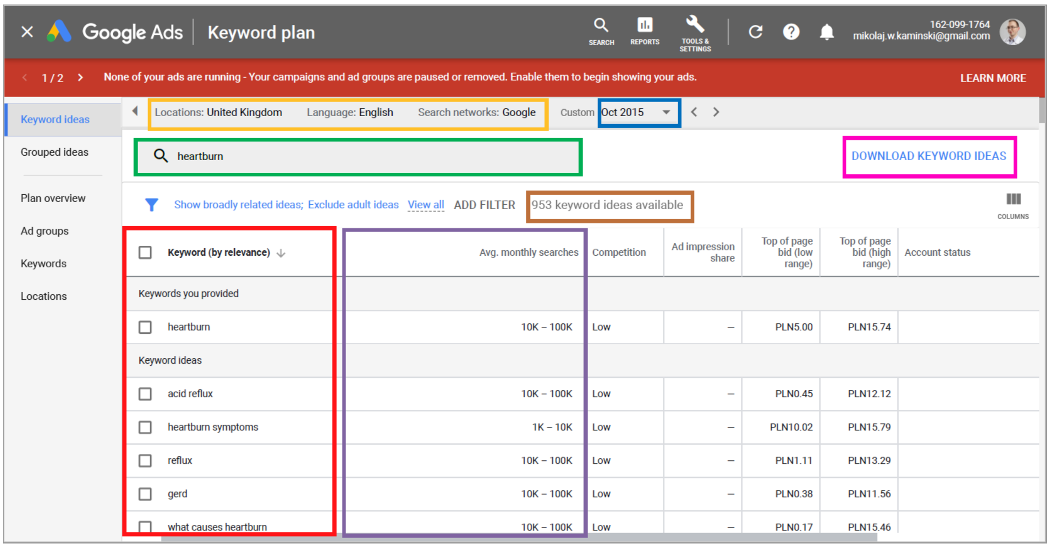

Figure S1. Screenshot of the keyword search engine of Google Ads Keywords Planner; The blue frame encapsulates the control for setting the time period of the search; brown frame – total number of related keywords found ("keyword ideas"); green frame – search bar for user-entered search terms; orange frame – control for setting the language and geographical region of the search, as well as the search network; pink frame – button for downloading a .csv file of the search results; red frame - long list of keywords; violet frame – monthly search volume for each keyword.

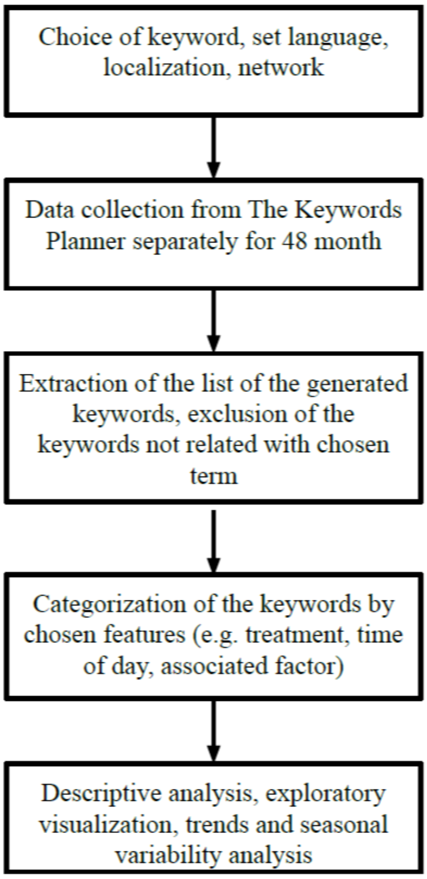

Figure S2. Flow chart of the data collection and manipulation workflow.
